# Saponins of North Atlantic Sea Cucumber: Chemistry, Health Benefits, and Future Prospectives

**DOI:** 10.3390/md21050262

**Published:** 2023-04-23

**Authors:** Oladapo F. Fagbohun, Jitcy S. Joseph, Olumayowa V. Oriyomi, H. P. Vasantha Rupasinghe

**Affiliations:** 1 Department of Plant, Food, and Environmental Sciences, Faculty of Agriculture, Dalhousie University, Truro, NS B2N 5E3, Canada; fg283429@dal.ca; 2 Department of Toxicology and Biochemistry, The National Institute of Occupational Health, A Division of National Health Laboratory Service, Johannesburg 1709, South Africa; 3 Department of Life & Consumer Sciences, University of South Africa, Johannesburg 1709, South Africa; 4 Department of Biological Sciences, First Technical University, Ibadan 200261, Nigeria; 5 Department of Pathology, Faculty of Medicine, Dalhousie University, Halifax, NS B3H 4H7, Canada

**Keywords:** sea cucumber, saponins, frondoside A, triterpenes, glycosides, *Cucumaria frondosa*

## Abstract

Frondosides are the major saponins (triterpene glycosides) of the North Atlantic sea cucumber (*Cucumaria frondosa*). Frondosides possess amphiphilic characteristics due to the presence of various hydrophilic sugar moieties and hydrophobic genin (sapogenin). Saponins are abundant in holothurians, including in sea cucumbers that are widely distributed across the northern part of the Atlantic Ocean. Over 300 triterpene glycosides have been isolated, identified, and categorized from many species of sea cucumbers. Furthermore, specific saponins from sea cucumbers are broadly classified on the basis of the fron-dosides that have been widely studied. Recent studies have shown that frondoside-containing extracts from *C. frondosa* exhibit anticancer, anti-obesity, anti-hyperuricemic, anticoagulant, antioxidant, antimicrobial, antiangiogenic, antithrombotic, anti-inflammatory, antitumor, and immunomodulatory activities. However, the exact mechanism(s) of action of biological activities of frondosides is not clearly understood. The function of some frondosides as chemical defense molecules need to be understood. Therefore, this review discusses the different frondosides of *C. frondosa* and their potential therapeutic activities in relation to the postulated mechanism(s) of action. In addition, recent advances in emerging extraction techniques of frondosides and other saponins and future perspectives are discussed.

## 1. Introduction

Sea cucumbers are nutrient-rich, invertebrate deep-sea or shallow-water dwellers used for centuries as an anti-inflammatory and anti-disease food source for treating various ailments [1]. They are marine organisms belonging to the phylum Echinodermata [2]. Almost 1500 sea cucumber species are discovered worldwide [3] and about 100 of them are well-known for human consumption [4], while more than 40 species of these are found to be edible [5]. The most important commercial species are *Apostichopus japonicus*, *Acaudina molpadioides*, *Actinopyga mauritiana*, *Cucumaria frondosa*, *Cucumaria japonica*, *Holothuria forskali*, *Holothuria scabra*, *Holothuria polii*, *Holothuria nobilis*, *Holothuria tubulosa*, *Isostichopus badionotus*, and *Pearsonothuria graeffei* [6]. Components of sea cucumbers possess different biological activities such as anticancer, anti-inflammatory, anticoagulant, hypolipidemic, wound healing, and hypoglycemic activities, all of which have obvious implications in the prevention and treatment of cardiometabolic diseases [7,8,9]. Interestingly, sea cucumbers are rich sources of several potent bioactive compounds, especially saponins, chondroitin sulfates, glycosaminoglycans, and sulfated polysaccharides [10,11,12].

SIn addition, several studies on sea cucumber have revealed that it is made up of many nutrients and bioactive constituents ranging from protein (mainly collagen), lipid (mostly omega-3 and omega-6 fatty acids), vitamins, trace elements, and minerals, mainly magnesium, zinc, calcium, and iron [13]. From a nutritional point of view, sea cucumber can be considered a balanced nutrient-containing food. It has high nutritional value because of its higher protein level and lower fat level than most other organisms. Furthermore, the protein profile of sea cucumbers is rich in lysine, arginine, and tryptophan [14]. Moreover, the gelatin from sea cucumber is considered to be more valuable than other gelatins because of its characteristic amino acid composition, especially the essential amino acids [15]. These include glycine, glutamic acid, and arginine, which have been reported to have a remarkable function in immune regulation [16,17,18]. Interestingly, saponins found in sea cucumbers are reported to be the basis of their chemical defense [19]. Even though saponins are found in plants, bacteria, fungi, and animals; however, there are differences in the saponins found in different sources based on the types and structures found in each group [20]. For example, saponins found in sea cucumbers are classified into several types based on their aglycone such as lanostane (including holostane and non-holostane) [21]. Many studies have reported that frondoside A is the major saponin in *C. frondosa* and it has an aglycone derived from holonostane as well as a sulfate group on its first sugar residue [22]. Interestingly, saponins in plants have their aglycone derived from either oleanolic acid or dammarane [23,24], while other organisms such as fungi, algae, and some invertebrates have saponins with aglycone derived from lanosterol [25]. In addition, it is worth noting that most saponins are either or non-sulfated triterpene glycosides [6]. Sea cucumber is widely used in traditional medicine for numerous nutritive and health benefits as well as used in the treatment of chronic inflammatory diseases [10,26,27]. In some parts of the world, sea cucumbers have long been used as traditional food and folk medicine for the treatment of hypertension, asthma, anemia, rheumatism, and sinus congestion [14,28]. Sea cucumber fucoidan has been reported to have anticoagulant, anti-hyperglycemic, anti-inflammatory, and immunomodulatory activity [29]. Growing evidence demonstrates that fucoidan possesses marked anticancer and anti-metastatic effects [30]. Out of the several species of sea cucumbers, *C. frondosa* has not been fully explored in terms of its therapeutic potential and bioactive constituents, especially the structural diversity and composition of saponins and other triterpene glycosides [6]. Therefore, this review discusses in detail the different classes of saponins found in *C. frondosa* in relation to their chemistry, health benefits, and future research perspectives. Moreover, specific saponins found in *C. frondosa* as well as the structure, chemistry, extraction techniques, biological activities, and potential mechanism(s) of action are reviewed while recent studies on gene expression and regulation of cell signaling pathways are discussed.

## 2. *Cucumaria frondosa*

**Figure 1:**
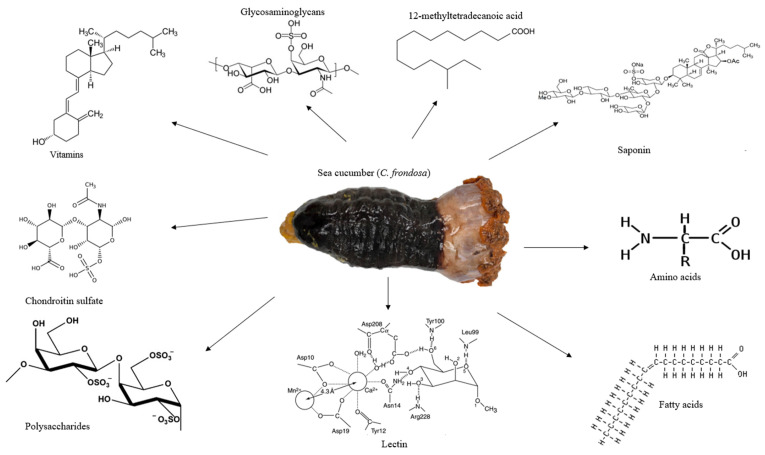
The appearance of sea cucumber (*C. frondosa*) and its major nutrients and bioactives. *C. frondosa* has been found to contain essential amino acids, glycosaminoglycans, vitamins, chondroitin sulfate, polysaccharides, lectin, and 12-methyltetradecanoic acid.

*C. frondosa* is one of the largest sea cucumbers and the widespread species of the *Cucumaria* genus, the Cucumariidae family in the Holothuroidea class (Figure 1). It is largely found in New England, the USA, North Atlantic Ocean, and Russia’s Barents Sea [31,32,33]. Several common names are attributed to *C. frondosa*, which include Atlantic sea cucumber in the USA, ‘brunpolse’ in Norway, ‘holothurie touffue’ in France, ‘Saebjuga’ in Iceland, and ‘Schwarze seegurke’ in Germany [34]. The orange-footed sea cucumber (*C. frondosa*) is the largest sea cucumber in the Atlantic Ocean and also one of the most commercial species [6]. Generally, sea cucumbers eat mud or dead particle remains; however, *C. frondosa* feed on phytoplankton, zooplankton, and organic matter by spreading out their tentacles [6,13]. *C. frondosa* species have a leathery skin texture and orange to black colored tentacles [6]. They can be about 20 cm long in size and mainly live in the deep part of the ocean in crevices and rock. The mouth is surrounded by aqua-pharyngeal bulbs/tentacles/flowers at one end of the body and an anus at the opposite end. They can grow to a maximum length of 40–50 cm, a width of 10–15 cm, and a weight of 100 to 500 g [6]. They control their movement with thousands of tiny tube feet and communicate with each other by transferring hormone signals through the water [35]. Generally, *C. frondosa* is harvested from May to November in Atlantic Canada with a growth rate slower compared with other sea cucumber species, with an average growth rate of 2 mm per month [6]. The Canadian landing of *C. frondosa* had about a 54.5% increase from 2008 to 2017 with a striking profit of CAD 18.3 million and accounted for a total revenue of about CAD 4 million to the Canadian economy [6].

Investigations on the nutritional and therapeutic benefits of *C. frondosa* have been receiving more attention in recent years. According to the comprehensive report by Hossain, Dave [6], *C. frondosa* are usually soft-bodied echinoderms, appear like a cucumber, and are a diverse group of flexible, elongated, worm-like organisms with leathery skin and a gelatinous body consisting of vitamins, minerals, cerebrosides, lectins peptides, and unique molecules such as 12-methyltetradecanoic acid, chondroitin sulfates, sulfated polysaccharides, triterpene glycoside compounds, and glycosaminoglycan (Figure 1) [28]. However, limited studies have been reported on the importance of saponins found in *C. frondosa*. Even though saponins play a major and crucial role in chemical defense, the majority of the studies found in the literature concluded that the main saponin in *C. frondosa* is frondoside A. Nevertheless, with the advancement in the extraction optimization and characterization through the use of high-performance liquid chromatography (HPLC)-time-of-flight mass spectrometry (TOF/MS), high-resolution mass spectrometry (HRMS), nuclear magnetic resonance (NMR), and other modern chromatographic techniques facilitated by data analysis using artificial intelligence (AI) software such as response surface methodology (RSM) and artificial neural network (ANN) mechanisms, several newer saponins can be isolated and characterized.

## 3. Saponins (Triterpene Glycosides) from *C. frondosa*

In general terms, saponins are the primary secondary metabolites of sea cucumbers, which are the basis of their chemical defense. Studies have reported that saponins extracted from sea cucumber show biological properties including antitumor [36], anti-obesity [37], anti-fungal, anti-bacterial [38], and anti-hyperuricemic activities and immune-modulatory activities [39]. Based on these impressive properties, several studies have isolated, analyzed, and characterized saponins from different organisms [1,6,8,10,13,40,41]. From their studies, they concluded that saponins are amphiphilic compounds composed of polar saccharide chains (hexose, pentose) attached to a non-polar (fat-soluble) aglycone [13]. The saccharide chain may be linear or branched and includes from 2 to 6 sugar units [41]. Saponins have a polycyclic ring system in their aglycone (either 27-carbon sterol or 30-carbon triterpene). The sugar moiety in a saponin molecule is attached to an aglycone by glycosidic linkage. They are glycosylated compounds or glycosides that are di-vided into three main groups according to the carbon skeleton of non-polar aglycone region: triterpenoidal glycosides, and steroidal glycosides [28]. Saponins that have been characterized in sea cucumbers are commonly identified as triterpene glycosides [42].

Saponins can be found in various body parts of holothurians, but they are not the same depending on the part. The distribution of saponins in the different parts of sea cucumbers such as the body walls, viscera, and Cuvierian tubules have been reported using both conventional matrix-assisted laser desorption/ionization (MALDI) and MALDI mass spectrometric imaging (MALDI-MSI) analyses [43,44]. The results of these studies revealed that the viscera of sea cucumbers have sulfated saponins and the sulfate groups are found on the xylose ring structure while the glycosides in some Cuvierian tubules and body walls have non-sulfated saponins. However, the major abundant saponins were sulfated congeners [21]. Holothurians also seem to secrete more saponins in stressful situations, and it is likely to be the case in this factory where they are cut while alive [9]. Saponins are mainly produced not only in planta, but rather marine organisms, animals, and bacteria [1]. Saponins found in *C. frondosa* are broadly classified on the basis of the different groups of frondosides. Several researchers have studied frondoside A and postulated the mechanism of action. To the best of our knowledge, this is the first comprehensive report on the different types of saponins found in *C. frondosa*.

### 3.1. Frondosides

**Figure 2:**
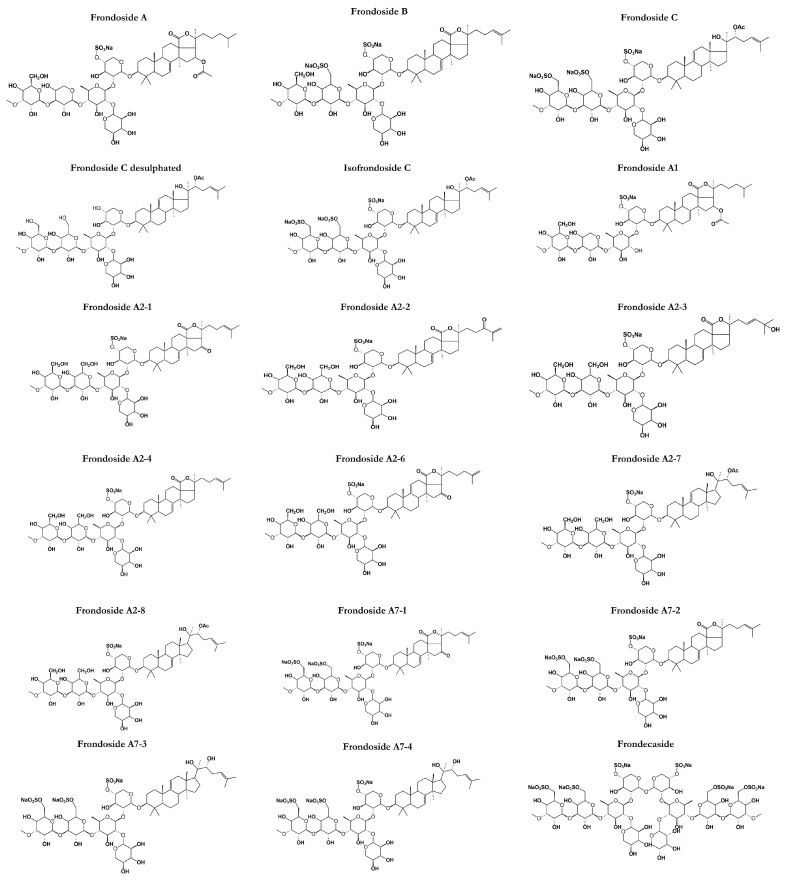
Structures of frondosides in sea cucumbers (*C. frondosa*) showing the polar saccharide chains (hexose, pentose, or uronic acid) attached with a non-polar (fat-soluble) aglycone. The saccharide chain includes one or more linear oligosaccharides that have chain lengths varying from 2 to 6 sugar units.

*C. frondosa* contains a mixture of mono-, di-, and tri-sulphated triterpene glycosides including frondoside A, frondoside B, frondoside C, isofrondoside C, frondoside A2-1, frondoside A2-2, frondoside A2-3, frondoside A2-4, frondoside A2-6, frondoside A2-7, frondoside A2-8, frondoside A7-1, frondoside A7-2, frondoside A7-3, and frondoside A7-4 (Figure 2) [22,35,45,46,47,48,49,50]. A study by Findlay and Yayli [47] reported that the major triterpene glycosides in *C. frondosa* is mono-sulfated frondoside A, which can be isolated from other components of the total glycoside fraction [47]. Moreover, di-sulfated frondoside B, and tri-sulfated frondoside C are found at slightly higher concentrations in *C. frondosa* [46,47,50]. There are only some minor structural differences between frondoside A, B, but frondoside C has nonholostane aglycone [51]. Other frondosides are E and F which are minor saponins (Figure 2).

### 3.2. General Overview, Chemistry, and Discovery

Frondoside A (C_60_H_95_NaO_29_S) (Figure 2) was discovered in 1990 as a triterpenoid glycoside of the lanosterol type with a distinctive D-ring fused γ-lactone and obtained as a colorless crystalline solid by high-perfomance liquid chromatography from the body walls of *C. frondosa* [51] at Quebec, Canada [50]. Frondoside A is a triterpenoid glycoside with an acetoxy group at C-16 in the aglycone (lanostane derivative). It is with 3-*O*-methylglucose as the terminal monosaccharide residue and xylose as the third monosaccharide residue as well as a sulphate group on the first sugar residue [52]. Frondoside A was the first saponin to be discovered from *C. frondosa* and has a molecular mass of 1334 Da while other frondosides have around the same molecular mass. Other types of frondoside A such as A1 and A2 (Figure 2) are detected as isomers of frondoside A. For example, frondanol A5 (lipid and saponin containing extract) was discovered as a novel therapeutic agent derived from sea cucumber, *C. frondosa* showing promising antiproliferative, anti-inflammatory, and antiangiogenic activities [53]. On the other hand, frondoside B, basing on ^1^H COSY, relay COSY, NOESY, and ^13^C-NMR data, was shown to have the structure 3β-*O*-{3-*O*-methyl-β-d-glucopyranosyl-(1→3)-*O*-β-d-6-sulfonatoglucopyranosyl-(1→4)-*O*-[β-d-xylopyranosyl-(1→2)}-*O*-β-d-quinovopyranosyl-(1→2)-*O*-β-d-4-sulfonatoxylopyranosyl}-holost-7-ene sodium (or potassium) salt. Standard 1D and 2D NMR procedures were employed to elucidate the structure.

Frondoside B alongside frondecaside was discovered when Findlay and Yayli [47] analyzed the whole specimens of *C. frondosa* collected from Passamaquoddy Bay, New Brunswick, Canada while frondoside C is a triterpene glycoside with the glycosides closed by aglycone, having a lanostane-type aglycone devoid of the typical 18(20) lactone ring and a sulfated non-holostane with the glycosides closed by aglycone structure as impurities. It is the seventh glycoside reported from the sea cucumber, *C. frondosa*. Frondosides E and F (Figure 2) were discovered in Canada as minor saponins from *C. frondosa*. Frondoside E was obtained as an inseparable mixture of E1 and E2. Frondoside E2 possesses an identical mono-sulfated pentasaccharide chain coupled via C-3 to a holosta-7,25-dione-16-one aglycone instead of the holosta-9,24-diene-16-one aglycone of E1 [54]. Frondoside F features a tri-sulfated pentasaccharide side chain coupled to modified holost-24-ene aglycone with a novel C-18-22 λ-lactone. These minor saponins are identical to another type of saponin from the *C. frondosa* species which is cucumarioside A2-2 isolated by Yayli [54].

### 3.3. Extraction and Detection of Frondosides

**Figure 3:**
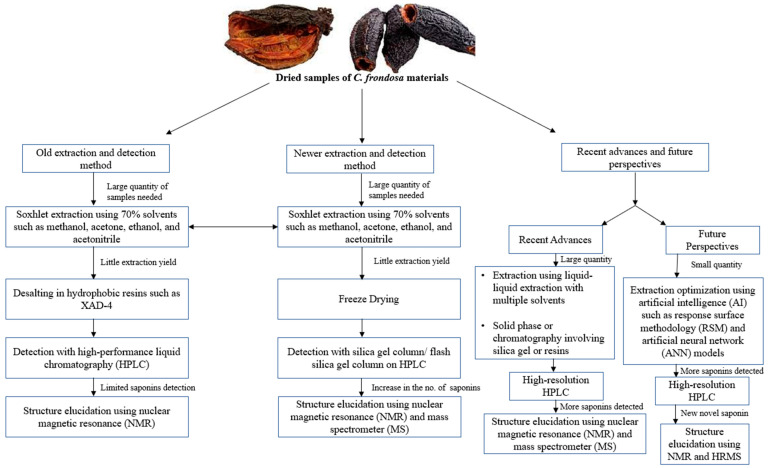
Summary of the extraction of sea cucumber (*C. frondosa*) materials for saponin detection over the last few decades. There were fewer saponins detected using the old extraction method while future perspectives focus on using little quantities of sea cucumber materials to detect new and novel saponins through the use of AI models such as response surface methodology (RSM) and artificial neural networks (ANNs).

Isolation of frondosides includes extraction and purification by segmentation chromatography methods (Figure 3). Isolation of triterpene glycosides from sea cucumbers is generally different in comparison with the triterpene glycosides from plants because of its polar characteristics; high proteins, lipids, peptides, and salts in the sea cucumber. Studies have reported the most common way of segregation of the glycosides including frondoside A is by extraction of crude animal material with 70% ethanol or methanol and desalting of the evaporated extract on hydrophobic resin namely XAD-4 followed by the isolation of individual glycosides on high-performance liquid chromatography (HPLC) [55,56]; however, the same method is not suitable for industrial purposes due to its colloidal character of the water solution of dried ethanol or methanol extract containing a lot of particles of various non-polar compounds.

Later, Avilov and colleagues invented a new method for the isolation of frondosides extracted from either the freeze-dried cooking water from the *C. frondosa* processing industrial plants or freeze-dried or powdered tissues of *C. frondosa* [57]. After extraction, the freeze-dried samples are dissolved in a mixture of chloroform and methanol, and thereafter, evaporation and followed by isolation. The evaporated extracts are dissolved in water and mixed with ethyl acetate to obtain an aqueous phase. After the phase separation, the frondosides are then purified on a common low-performance silica gel column or using the simplest flash silica gel column on common chromatography. The major component of the final purification fraction contains frondosides [57]. The purity of the extracted frondosides can be detected by ^13^C NMR and mass-spectrometry. Previous studies also reported the purification of frondosides using various techniques including liquid–liquid extraction with multiple solvents, HPLC, solid-phase extraction, or chromatography (resins or silica gel) [48,50]. For the determination of novel saponins, newer extraction methods should be employed as shown in Figure 3.

### 3.4. Pharmacological Relevance

Frondosides have a wide range of pharmacological properties. Frondoside A has been extensively studied among all the different types of frondosides found in *C. frondosa*. Frondoside A present in *C. frondosa* was reported to possess immunomodulatory properties when administered in subtoxic doses. A study conducted by Aminin and Agafonova [58] indicated that the lysosomal activity of mouse macrophages was stimulated by the administration of a 0.2 μg dose of frondoside A and maintained over 10 days in an in vivo study. Moreover, the lysosomal activity of mouse macrophages was also stimulated by the administration of 0.1–0.38 μg/mL dose of frondoside A in vitro. The same study also shows that frondoside A improves macrophage phagocytosis at a maximally effective dose of 0.001 µg of the bacterium, *Staphylococcus aureus*, in vitro. Frondoside A also increases reactive oxygen species (ROS) generation at a maximally effective concentration of 0.001 μg in macrophages in vitro [59]. Other studies have also reported that frondoside A is beneficial in stimulating the immune function at a dose less than the dose needed for inhibiting cancer cell growth in mammalian splenocytes [51,60]. Nevertheless, a study by Janakiram and Mohammed [28] showed that frondoside A increases innate immune responses followed by inhibition of intestinal tumor growth in mice. Together, these studies indicate the potential immunomodulatory effects of frondoside A where it may provide preventive treatment benefits against diseases wherein a reduced immune status contributes to the pathological processes. Frondoside A has potent anti-invasive, anti-proliferative, and anti-angiogenic effects on several cancers including adenocarcinomas of the breast, lung, colon, prostate, leukemia, and pancreatic cancers [61,62,63].

Studies have shown that frondoside A is a potential compound to target multiple cancer cell characteristics used in cancer therapy by inhibiting cancer cell growth, migration, invasion, formation of metastases, and angiogenesis [52]. Frondoside A treatments have minimal toxic effects on normal cells, while cancer cells including pancreatic cancer, lung cancer, colon cancer, and prostate cancer cell lines are specifically targeted [52]. Furthermore, frondoside A is shown to result in a concentration-dependent decrease in the viability of hepatoma cells (HepG2) and lung cancer cells; LNM35, A549, and NCI-H460-Luc2, breast cancer cells (MCF-7), and melanoma cells (MDA-MB-435) over 24 h, and elevated the activities of caspases-3 and -7 in LNM35 lung cancer cell [64]. A study was conducted using various human prostate cancer cell lines including PC-3, DU145, and VCaP. The 22Rv1 and LNCaP reported that frondoside A causes a decrease in colony formation and cell viability compared with normal cell lines [65]. Its properties include simultaneous induction of apoptosis in combination with cell cycle arrest, potential immune modulatory effects, and inhibition of pro-survival autophagy making the compound a favorable candidate for the treatment of prostate cancer.

A study conducted by Attoub and Arafat [11] confirmed the ability of frondoside A to suppress lung cancer growth in vivo where frondoside A inhibits the growth of A549 lung cancer cells with IC_50_ ranging between 1 and 3 μM in cell culture and upregulation of the tumor suppressor p21, an inhibitor of cyclin-dependent kinases (CDKs). It is also reported that a combination of frondoside A and butein causes a concentration- and time-dependent decrease in the viability of the lung cancer cells including A549 and LNM35 [66]. Another recent study also demonstrated that the combination of butein with frondoside A results in the inhibition of A549 and cellular viability, induction of caspase 3/7 activity, inhibition of colony growth and cellular migration, and invasion [67]. Moreover, frondoside A is known to inhibit the growth of pancreatic cancer cells by promoting apoptosis via a cascade of activation of mitochondrial pathways [63]. A combination of frondoside A and gemcitabine is suggested to be more effective against pancreatic cancer compared with a single dose [68].

Interestingly, there are few studies that have compared the efficacy of different types of frondosides based on their pharmacological properties. It was demonstrated in the reports of Sajwani [15] and Al Shemaili and Parekh [69] that frondoside A has a greater growth inhibitory effect on the pancreatic cancer cell lines AsPC-1 and S2-013 compared with frondoside B and C. Studies have also reported the higher antiangiogenic and antimetastatic effects on the cancer inhibitory effect using tumor-bearing mice than other types of frondosides [62,63,64]. Recently, Ru and Chen [70] investigated the individual anti-bladder cancer effects of both frondoside A and its combination with CpG oligodeoxynucleotide (CpG-ODN) in vitro and in vivo. From the results of the study, it was revealed that frondoside A has a potential anti-bladder cancer activity by inhibiting cell viability and migration, inducing apoptosis, and affecting the cell cycle. Therefore, the combined therapy of CpG-ODN with frondoside A can be a promising approach to treating bladder cancer (Table 1).

### 3.5. Mechanism(s) of Action of Frondosides

**Figure 4:**
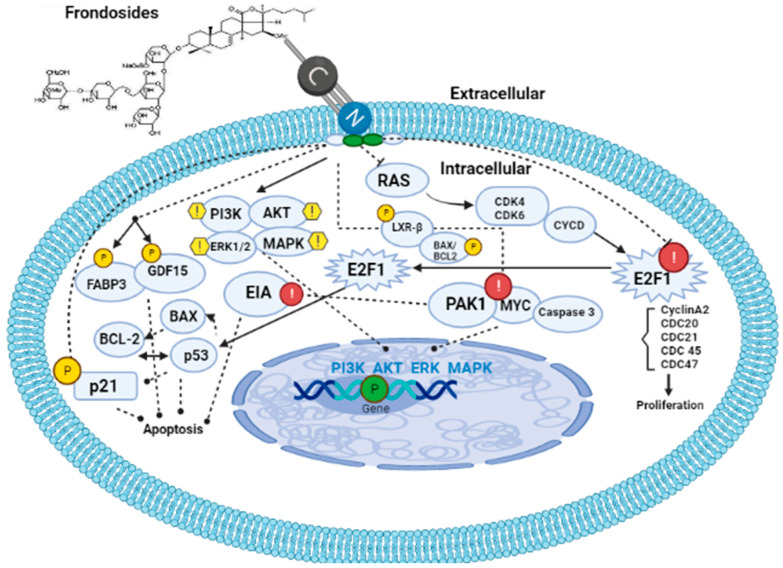
Mechanism of action of frondosides. Frondosides interact with the cell membrane via their phospholipid bilayer leaving a hole. Once inside the cell, frondosides tend to regulate PI3K/AKT/ERK1/2/MAPK signaling pathways as well as upregulate p21, FABP3, and GDF15 genes, thereby leading to the inhibition of metastasis and angiogenesis in the cancer-affected organism. Frondosides also inhibit and downregulate E2F1(cyclin A2, CDC 20, 21, 45, and 47), PAK1, MYC, and Caspase 3. PI3K: phosphatidylinositol 3-kinase; MAPK: mitogen-activated protein kinases; FABP3: fatty acid binding protein 3; GDF15: growth and development factor 15.

To date, the mechanism(s) of the action of frondosides, specifically frondoside A, has been a subject of debate, but a large number of studies have linked the mechanism, underlying its action to the steroid nucleus of the molecule in comparison with the actions of steroidal anti-inflammatory drugs (SAIDs) while some other sources have postulated the mechanism as inhibition of cell growth, survival, migration, invasion, metastasis, and angiogenesis in cancer therapy. These discoveries have tended towards the upregulation of fatty acid binding protein 3 (FABP3), which is a tumor suppressor that arrests the growth of mammalian epithelial cells as well as the upregulation of growth and development factor 15 (GDF15) which belongs to the transforming growth factor superfamily that plays a role in regulating inflammatory and apoptotic pathways during tissue injury and mediates apoptosis induction in response to non-steroidal anti-inflammatory drugs (NSAIDs) [68,69]. Moreover, the same study also discovered that frondosides also downregulate some genes involved in DNA replication and cell cycle control such as E2FI, cyclin A2, CDC20, CDC21, CDC45, and CDC47 as well as the repression of dual-specificity phosphatase and death associated protein kinase (EIA) [68]. A study on the knockdown of expression of either GDF15 or FABP3 using specific siRNA in AsPC-1 cells reversed the growth inhibitory effects of frondosides (Figure 4).

Park and Kim [79] revealed that frondosides can inhibit tissue plasminogen (TPA)-induced activation of matrix metalloproteinase-9 via pathways involving inhibition of activation of two transcriptional factors (AP-1 and NF-κB) as well as the reduction in ATP-stimulated phosphorylation of several kinase pathways such as PI3K/AKT/ERK1/2/p38 MAPK (Figure 4). Furthermore, Nguyen and Yoshimura [71] reported that frondosides inhibited RAC/CDC42-activated kinase (PAK1) with an inhibitory concentration (IC_50_) around 1.2 µm in vitro (Figure 4). PAK1 is a kinase enzyme that inhibits cell growth, invasion, and metastasis by suppressing the expression of p21 gene. Moreover, upregulation of BAX, several anti-apoptotic proteins, cleavage of PARP, caspase 3 as well as downregulation of anti-apoptotic proteins such as survivin and BCL2 were reported by Pislyagin and Manzhulo [39], Dyshlovoy and Otte [65], and Dyshlovoy and Menchinskaya [80]. Recent studies have found that frondosides suppress MYC expression and its gene targets in the medulloblastoma model derived from human-induced pluripotent stem cells [81]. This study created an awareness of the therapeutic effects of saponin in cancer therapy since MYC is a super transcriptional factor whose expression is upregulated in over 70% of cancer types and it is known that MYC is the hallmark of cancer initiation and maintenance. Finally, the anti-obesity effects of saponin were reported to be a result of the upregulation of LXR-β signaling and inhibition of pancreatic lipase activity by the study of Guo and Gao [82] on saponin-enriched extracts of sea cucumbers (Figure 4).

### 3.6. Future Perspectives

It is believed that the secondary metabolites of sea cucumbers are numerous and yet to be fully explored as a result of extraction methods and detection techniques. To date, there is no report that describes the optimization of the extraction of saponins from *C. frondosa*. This creates a gap in research regarding the discovery of secondary metabolites in *C. frondosa*. Our laboratory is currently exploring the use of an artificial intelligence model to optimize the extraction of sea cucumber materials for the detection of novel saponins. This is made possible through the use of RSM and ANN. Moreover, the majority of the study on saponins in sea cucumbers were carried out on the body walls [6]. However, the report by Van Dyck and Gerbaux [25] concluded that saponin contents were always more concentrated and 11-fold higher in the internal organs of sea cucumbers than in the body walls. From the results of their study, it was revealed that the concentrations of saponin in the internal organs ranged from 1.38 to 11.36 mg/g (wet weight) while that of the concentrations in the body walls ranged from 0.32 to 2.40 mg/g (wet weight). This creates a research gap and confusion in the scientific literature regarding the content of saponins in different parts of *C. frondosa*. It is worth noting that much of the research on these compounds is in its early stages and more research is needed on the extraction methods, pharmacology, and mechanism(s) of action to fully understand their potential health benefits. Additionally, the extraction and purification of these compounds can be complex and may require specialized equipment and expertise. Furthermore, recommendations can be made on the use of extraction optimization techniques such as RSM and ANN which have not been reported in the literature for extraction of newer and maybe novel saponins from sea cucumber materials.

## 4. Cucumariosides

**Figure 5:**
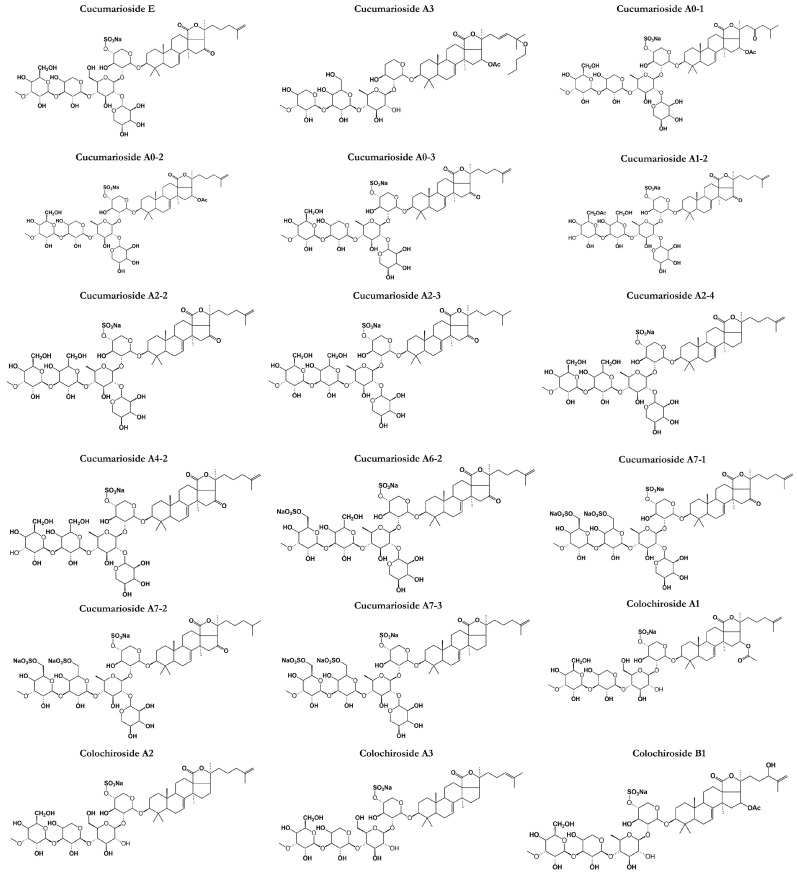

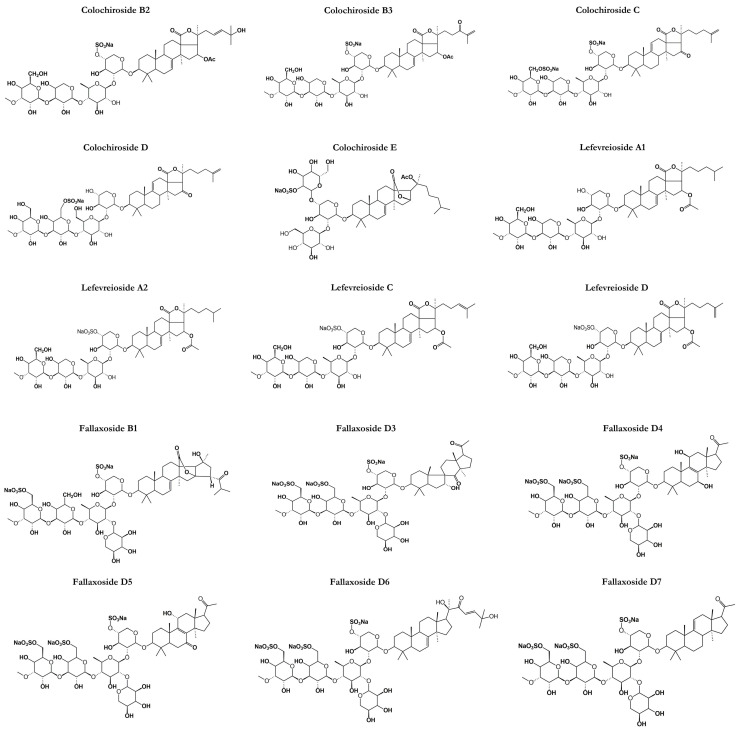
Chemical structures of other classes of saponins present in sea cucumbers.

Cucumariosides are a type of triterpene glycoside that has been identified in *C. japonica* and *Eupentacta* (=Cucumaria) *fraudatrix* known to produce a variety of cucumariosides [83]. Cucumariosides are known to exhibit various pharmacological activities, including antitumor, antiviral, and immunomodulatory effects (Figure 5). They have been studied extensively for their therapeutic applications and have been shown to inhibit the growth of cancer cells, modulate the immune system, and exhibit antiviral activities against several viruses [83].

### 4.1. General Overview, Chemistry, and Discovery

The discovery of cucumariosides dates back to the early 20th century when researchers first isolated these compounds from the body walls of various sea cucumber species [84]. Since then, hundreds of cucumariosides have been identified and characterized from different species of sea cucumbers, including *C. frondosa* [76]. The identification of new cucumariosides has been facilitated by the development of advanced analytical techniques, such as HPLC, HRMS, and NMR spectroscopy [85]. Recent studies have also employed metabolomic and genomic approaches to identify and characterize new cucumariosides from sea cucumbers, which has provided insights into the biosynthesis and diversity of these compounds. Overall, the discovery of new cucumariosides is an active area of research for scientists, with potential applications in drug discovery and development [86].

While cucumariosides have not been widely studied nor received the scientific attention needed, there are about four different triterpene glycosides of cucumarioside types that have been greatly isolated with many pharmacological properties. Cucumarioside A2-2 is a triterpene glycoside composed of a lanostane-type aglycone having 18(20)-lactones belonging to the holostane types.

### 4.2. Pharmacological Relevance and Mechanism of Action

Cucumariosides have been shown to exhibit antitumor activity by inducing apoptosis in human leukemia cells as well as inhibiting the growth of colon cancer cells [7]. Moreover, recent studies have further discovered the anticancer effects of cucumariosides in melanoma and breast cancer cells [87]. There is evidence that cucumariosides have potential applications as an anti-viral agent after it was discovered that they inhibit virus replication by inducing interferon production while the immunomodulatory activities have been reported as a result of their activities in the enhancement of the activity of natural killer cells by stimulating the production of cytokines [88]. The mechanism of action of cucumariosides is not completely understood, but it is involved in multiple pathways. For example, cucumariosides have been shown to induce apoptosis in cancer cells by activating caspase-dependent and -independent pathways. They have also been shown to inhibit the growth of cancer cells by disrupting the cell cycle and inhibiting cell proliferation. In addition, cucumariosides have been shown to modulate the immune system by enhancing the activity of killer cells, thereby stimulating the production of cytokines [35,75,89,90].

### 4.3. Future Perspectives

Overall, cucumariosides are an important class of bioactive compounds found in sea cucumbers, which have potential applications in the development of new drugs and therapies. Further research is needed to fully understand their mechanisms of action and the specific signaling pathways that they regulate to fully explore their potential therapeutic uses [16,26].

## 5. Application of RSM and ANN for the Optimization of Extraction of Saponins from *C. frondosa*

The extraction of saponins from *C. frondosa* is a complex process that requires optimization to ensure the maximum yield and quality of the extract [16,91]. RSM and ANN are two popular optimization techniques that can be used to determine the optimal conditions for saponin extraction from *C. frondosa* [92,93], and this can be the new mechanism for the discovery of potentially new saponins that have not been reported before in scientific literature. RSM is a statistical technique that involves the use of mathematical models to identify the relationship between independent variables and a response variable [94]. In the case of saponin extraction, the independent variables can include factors such as extraction temperature, extraction time, solvent type, and concentration, and sample-to-solvent ratio. The response variable can be the yield or quality of the saponin extract. RSM involves the design of experiments to obtain data at different levels of the independent variables. These data are then used to construct a model that predicts the response variable as a function of the independent variables. The model can be used to identify the optimal conditions for saponin extraction [95,96].

On the other hand, ANN is a computational technique that involves the use of artificial neural networks to identify patterns in data. ANN has been shown to be an effective optimization technique for complex processes such as saponin extraction. In the case of saponin extraction from *C. frondosa*, ANN can be used to identify the optimal combination of independent variables that result in maximum yield and quality of the saponin extract. ANN involves the use of a network of interconnected nodes that process data and identify patterns. The ANN model can be trained using data obtained from experimental designs at different levels of the independent variables. Once the ANN model is trained, it can be used to predict the optimal conditions for saponin extraction [97].

## 6. Conclusions

The North Atlantic sea cucumber contains several different types of saponins with potentially interesting therapeutic properties. These saponins contain a lipid-like aglycone and a carbohydrate chain attached via glycosidic linkage. The aglycone por-tion of the saponin can vary depending on the specific compound. In general, they tend to have a triterpene or steroid nucleus and this forms the basis of their pharmacologi-cal activities. However, there are a lot of unidentified or characterized saponins that can be discovered from the least explored sea cucumber, *C. frondosa*, as explained in our review by improving of the extraction method. Despite the potential health benefits of these saponins, further research is needed to fully understand their biological activities and potential applications. For example, further research is needed to determine the optimal dosage and delivery methods for these compounds, as well as to assess any adverse effects. Understanding the efficacy and pharmacology of frondosides can lead to the development of novel functional foods, natural health products, drug leads, and cosmeceutical ingredients. In conclusion, RSM and ANN are powerful optimization techniques that can be used to determine the optimal conditions for saponin extraction from *C. frondosa*. These techniques can help in future research to improve the yield and quality of saponin extracts, which may have numerous potential health benefits.

## Figures and Tables

**Table 1:** Summary of the potential health benefits of saponins (triterpene glycosides) from different *Cucumaria* species.

Saponin	Sources	Extraction	Chemistry	Health Benefits	Pharmacology	Mechanism of Action	References
Frondosides	*C. frondosa*	Water extraction, ethanol precipitation	Composed of a trisaccharide and a steroid	Antioxidant, anti-inflammatory, anticancer	Neuroprotective effects and help reduce inflammation and oxidative stress.	Modulate the activity of inflammatory enzymes and reduce oxidative stress through its antioxidant properties.	[51,63,68,69,71,72]
Fallaxosides	*Cucumaria fallax*, a close relative of *C. frondosa*.	Methanol extraction, silica gel column chromatography	Contain a trisaccharide and a steroid	Anticancer, Anti-inflammatory	Anticancer activity and may help reduce inflammation	Induce apoptosis in cancer cells and reduce the production of pro-inflammatory cytokines	[73]
Colochirosides	*Cucumaria colochoi*, a close relative of *C. frondosa*	Methanol extraction, silica gel column chromatography	Composed of a sugar and a triterpene aglycone	Anticancer	Potent anticancer activity against various cancer cell lines	Inhibit cell proliferation and induce apoptosis in cancer cells	[74]
Cucumariosides	Various sea cucumbers including *C. frondosa*	Water or ethanol extraction, column chromatography	Composed of a sugar and a triterpene aglycone	Antioxidant, Immunomodulatory	Antioxidant and immunomodulatory effects, and potential to treat conditions such as arthritis	Modulate immune cell activity and reduce oxidative stress through its antioxidant properties	[39,61,75,76]
Lefevreiosides	*Cucumaria lefevrei*, a close relative of *C. frondosa*	Ethanol extraction, column chromatography	Consist of a trisaccharide and a steroid	Anticancer, Anti-inflammatory	Anticancer activity and may help reduce inflammation	Induce apoptosis in cancer cells and reduce the production of pro-inflammatory cytokines	[77,78]

## Data Availability

Not applicable.
